# Perinatal manifestation of mevalonate kinase deficiency and efficacy of anakinra

**DOI:** 10.1186/s12969-016-0081-9

**Published:** 2016-03-25

**Authors:** Skaiste Peciuliene, Birute Burnyte, Rymanta Gudaitiene, Skirmante Rusoniene, Nijole Drazdiene, Arunas Liubsys, Algirdas Utkus

**Affiliations:** Neonatology Centre of Vilnius University, Santariškių St. 7, Vilnius, Lithuania; Department of Human and Medical Genetics, Faculty of Medicine, Vilnius University, Vilnius, Lithuania; Pediatrics Centre of Vilnius University, Vilnius, Lithuania

**Keywords:** Mevalonate kinase deficiency, Mevalonic aciduria, Hyperimmunoglobulinemia D syndrome, Autoinflammatory syndrome, IL-1 antagonist

## Abstract

**Background:**

Mevalonate kinase deficiency is a metabolic autoinflammatory syndrome caused by mutations in the *MVK* gene, mevalonate kinase, the key enzyme in the non-sterol isoprenoid biosynthesis pathway. Two phenotypes of mevalonate kinase deficiency are known based on the level of enzymatic deficiency, mevalonic aciduria and hyperimmunoglobulinemia D syndrome, but a wide spectrum of intermediate phenotypes has been reported. Currently one of the most effective treatments is biological therapy (with interleukin-1 antagonist anakinra or tumour necrosis factor-α inhibitor etanercept).

**Case presentation:**

The patient in this case has a phenotype contributing to a severe disease that caused the symptoms to manifest very early, in the prenatal period. Mevalonate kinase deficiency was suspected on the basis of clinical (*hydrops fetalis*, hepatosplenomegaly, hypotonia) and laboratory signs (anaemia, intense acute phase reaction, increased urinary excretion of mevalonic acid). Mutation analysis of the *MVK* gene confirmed the biochemical diagnosis. Treatment with the interleukin-1 antagonist anakinra was started (minimal dose of 1 mg/kg/day) and revealed its efficacy after three days.

**Conclusions:**

Our case highlights the need for a very detailed clinical and laboratory assessment in new-borns with any suggestion of autoinflammatory disorders. It is important that patients are diagnosed as early as possible to provide better multidisciplinary follow-up and therapy when needed.

## Background

Mevalonate kinase deficiency (MKD) is a rare inborn error of metabolism caused by deficiency in the enzyme mevalonate kinase [1, 2]. It disrupts synthesis of cholesterol and non-sterol isoprenoids [1–7]. There are two forms of mevalonate kinase deficiency: mevalonic aciduria (MA), when residual function of the enzyme is undetectable (<1 % [3] or <0.5 % [8] according to different authors) and hyperimmunoglobulinemia D syndrome (HIDS), when residual function of the enzyme is 1.8–28 % [3, 9] (or 1–10 % [8]). Literature reveals about 30 cases of MA, while the milder form, HIDS, is ten times more common [2, 5, 10]. There is evidence that phenotypes of MA and HIDS overlap; patients with the HIDS phenotype develop neurologic symptoms later in life [11–13].

The pathogenesis of the disease is not definitely defined. Currently persistent systemic inflammation is thought to be the main pathogenic factor of the disease. The inflammation originates from impaired synthesis of non-sterol isoprenoids, namely farnesylpyrophosphate and geranylgeranylpyrophosphate that are used for protein prenylation [1, 3, 13]. The lack of prenylation leads to changed localization and functional activity of some proteins, e.g. overactivity of small GTPases. As a result inflammasome complexes are formed that activate caspase-1 and hypersecretion of IL-1β is mediated. There is data that inflammasome can also be activated by reactive oxygen species and mitochondrial dysfunction [3]. In mevalonic aciduria it is assumed that disruption in cholesterol synthesis mostly influences brain development [1, 10]. Exogenous cholesterol obtained with food meets the needs of steroid hormones, biliary acid synthesis, etc. but does not pass the blood–brain barrier. Therefore, neuronal myelinisation and the stability of the brain cell membrane is disturbed, and neurologic symptoms develop.

Although the clinical expression of mevalonate kinase deficiency can vary, the disease generally starts in infancy [2, 3, 5, 7, 10]. For the milder form (HIDS), the most specific symptom is recurrent episodes of fever with or without lymphadenopathy, arthralgia, myalgia, abdominal pain, diarrhoea, vomiting, polymorphic skin rash, and mucosal ulcers [2, 3, 5, 7–11, 14]. In some severe cases of mevalonic aciduria, *hydrops fetalis* and intrauterine growth restriction are observed. MA phenotype include hepatosplenomegaly, cholestatic jaundice, diarrhoea, vomiting, mild dysmorphic features (dolichocephaly, microcephaly, triangular-shaped face, down-slanting eyelids, dysplastic ears), and failure to thrive [2, 3, 5, 9–11, 14]. Hypotonia is observed from birth and cerebellar ataxia progresses during the course of the disease; psychomotor development may differ [2, 3, 5, 7, 10, 11]. Ocular impairment, such as cataract, uveitis, retinitis, can be identified in the course of the disease [2, 3, 5, 10, 11]. A wide spectrum of intermediate phenotypes has been reported [13].

Laboratory tests show intense acute phase reaction, anaemia, direct hyperbilirubinemia, and increased urinary excretion of mevalonic acid which can persist in MA patients and occurs during febrile attack in HIDS patients [3, 5, 7, 8, 10, 11]. Two-thirds of patients have elevated serum IgA and IgD [3, 10]. Diagnosis is confirmed by the genetic mutation being identified for patient and parents [3, 5, 10]. Prenatal diagnosis is possible (if there were cases in the family). After an amniocentesis or chorionic villous biopsy is performed, the activity of mevalonate kinase or the presence of mutations in the mevalonate kinase (*MVK*) gene can be determined [2, 5, 10]. Preimplantation genetic diagnosis should be discussed in families affected by a particularly severe form of MKD. Prenatal diagnosis and preimplantation genetic diagnosis should be performed after genetic counselling [15].

The aim of this case report is to describe the patient’s medical history between the first prenatal symptoms and the diagnosis of MKD and report the efficacy of timely treatment with the IL-1 antagonist anakinra.

## Case presentation

**Fig. 1:**
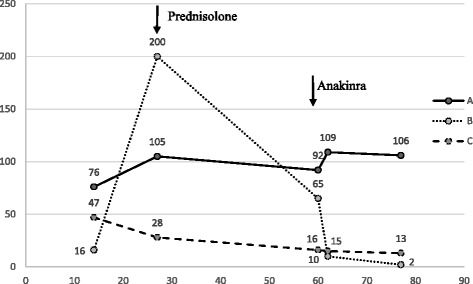
Laboratory values. The figure demonstrates dynamics of the laboratory values (**a** – Hgb g/l, **b** – CRP mg/l, **c** – WBC *10e9/l) before treatment and after treatment with systemic corticosteroids (on the 27^th^ day of life) and anakinra (on the 60^th^ day of life) was started

The patient is a male infant born to healthy non-consanguineous Lithuanian parents. The mother’s age at delivery was 32 years old, and this was her first pregnancy. From the 31^st^ week of pregnancy, she started to complain about pain in her left flank. Acute pyelonephritis was suspected and antibiotics were prescribed, but the urine culture was negative. The foetal ultrasound in the 31^st^ week was normal. Since the pain deteriorated, on the 33^rd^ week ultrasound was repeated and revealed foetal ascites, hydrocele, hepatosplenomegaly, moderate anaemia, tricuspid regurgitation, and polyhydramnios. Because of the critical foetal condition, an urgent C-section was performed. The male infant weighed 2 676 g and had a height of 48 cm and occipital-frontal circumference of 33.5 cm. The Apgar score was 8–8. Immediately after birth, hepatosplenomegaly, ascites and hydrothorax were observed. In the first week of life, a diffuse maculopapular rash appeared. Laboratory data showed severe anaemia, intense acute phase reaction, direct hyperbilirubinemia, elevated liver enzymes, metabolic acidosis, and hypokalaemia. TORCH titters, as well as blood and spinal fluid cultures, were negative. From the first hours of life, the new-born was treated with red blood cell transfusions, empiric antibiotics, sodium bicarbonate, and ursodeoxycholic acid. There was no therapeutic response to empiric antibiotics. On the 7^th^ day of life, feeding problems, lethargy, and hypotonia were observed. At the end of the 2^nd^ week, bloody diarrhoea, failure to thrive, and short (1–2-day) recurrent febrile episodes started. Screening for metabolic disorders performed on the 16^th^ day revealed increased urinary excretion of mevalonic acid, which is characteristic of mevalonate kinase deficiency. Given a presumed diagnosis of MKD, on the 9^th^ week of life he began to be treated with the IL-1 antagonist anakinra (1 mg/kg/day) on a daily basis. After a few days of treatment, the boy became more active, his temperature remained normal, his diarrhoea ceased, pronounced weight gain was observed (at first the patient gained 200 g in 2 months and after anakinra he gained 600 g in 3 weeks), and systemic inflammatory reaction markers and haemoglobin returned to normal (Fig. 1). Mutation analysis of the *MVK* gene revealed that the boy was homozygote for the nucleotide substitution c.1162C > T, leading to the aminoacid R388 change to a stop codon (p.R388Ter). The nonsense mutation has already been reported in the Infevers database at http://fmf.igh.cnrs.fr/ISSAID/infevers/ and also identified in other patients of Eastern Europe origin [16]. On follow-up at the chronological age of 4 months (adjusted age of 2 months), the boy was thriving but the weight was on the 3^rd^ percentile, he had no febrile attacks, and according to the mother every month he had intermittent periods of abdominal pain and diarrhoea that lasted for a week. The patient made eye contact, smiled back, and babbled but his head control was insufficient. On the examination at the chronological age of 7 months monthly periods of diarrhoea persisted, failure to thrive was observed – weight was less than 3^rd^ percentile. There were no febrile attacks. Motor development was impaired: the patient didn’t sit, head control was insufficient and hypotonia remained though mental development was normal. At the age of one year the patient had severe febrile attack with bloody diarrhoea after routine immunization, weight gain was extremely low. He developed normal head control, crawled, sitted with self support, played with toys. The dosage of anakinra was increased to 2 mg/kg/day and prednisone (2 mg/kg/day) was added to treatment. After 3 months of this treatment the patient was thriving better, periods of diarrhoea were less severe. Brain MRI, performed at age 1 years and 4 months, showed no structural abnormalities. Patient’s motor development have improved after intense rehabilitation, however it is still significantly delayed: he stands up and walks with considerable support, hypotonia remains (more pronounced in legs). The boy shows interest in the surroundings, communicates willingly, speaks with solitary words, builds a tower of blocks.

## Conclusions

The patient’s disease in this case report presented in the perinatal period, and due to its progression an emergency C-section was performed. Such early manifestation of the disease usually leads to a severe course and poorer outcome. Molecular genetic testing shown the previously reported homozygous mutation c.1162C > T of the *MVK* gene. Until 2012, 103 different mutations within the *MVK* had been described [3]. MKD is inherited in an autosomal recessive manner. A majority of the patients have compound heterozygous mutations but homozygous mutations have also been detected [3, 5, 10]. It has been noted that with a homozygous mutations the course of the disease is more severe and prognosis is poorer [3]. The phenotype of our patient was caused by a homozygous mutation in *MVK* gene and manifested in perinatal period. Thus, after evaluation of disease progression we conclude that the phenotype is of intermediate severity.

The diagnosis was made in a timely manner compared with data in the literature (sometimes it takes years to decades [11, 14]). It is important because the undiagnosed disease impact quality of life of the patient and parents also aggravate the work of the health care system (multiple hospitalisations, unnecessary exams and treatments) [14]. However secondary impairment of uncontrolled inflammation, such as amyloidosis, in MKD is rare [3, 14, 17]. There was a multicenter study including 18 countries performed and amyloidosis was found in 2,9 % of HIDS patients [17].

We could hypothesize that symptoms of the mother‘s pyelonephritis were caused by mevalonic acid excretion since there are cases described [5]. However there is no data in the past literature about treating the pregnant woman. The patient was treated with systemic corticosteroids (methylprednisolone) and later with anakinra as soon as the diagnosis of mevalonate kinase deficiency was suspected. According to the past literature, recurrent episodes of fever were treated with non-steroid anti-inflammatory drugs and systemic steroids, but results were disappointing [3–5, 9, 10]. Currently one of the most effective treatments is biological therapy [2, 4, 6–9, 11]. The medications of choice are the TNF-α inhibitor etanercept and the IL-1 antagonist anakinra*.* Based on Caorsi et al., treatment is ineffective in 43 % of cases with etanercept and in only 18 % of cases with anakinra [4]. The recommended dose of anakinra varies from 1 to 5 mg/kg/day. In our case, the minimum dose of 1 mg/kg/day was effective at the beginning of the disease. However, during the course of the disease inflammation of gastrointestinal tract persisted and the dose was increased. The new anti-IL-1 receptor antagonist canakinumab has showed promising results in five patients [9]. Its main advantage is extended exposure time and administration every 8 weeks. Nevertheless biological therapy is effective for the systemic inflammation it does not affect neurological symptoms. As mentioned earlier, it is assumed that brain development is mostly influenced by the disruption in cholesterol synthesis [1, 10]. Exogenous cholesterol does not pass the blood–brain barrier and therefore, neuronal myelinisation and the stability of the brain cell membrane is disturbed. Two cases of allogeneic bone marrow transplantation when biological therapy was not efficient are described in literature [7, 10]. Neven et al. presented a patient who had mevalonate kinase function in lymphocytes up to 64 %, no episodes of fever, and no progression of neurological symptoms (ataxia level remained the same as before transplantation) 15 months after transplantation [7].

Our case highlights the need for a very detailed clinical and laboratory assessment of new-borns with any suggestion of autoinflammatory disorders. Further investigation such as metabolic screening should be considered in every patient with persistent inflammatory markers with negative cultures. It is therefore important for patients to be diagnosed as early as possible to provide better multidisciplinary follow-up and therapy when needed. It is important for health care professionals to collect and publish long term information about the course and treatment of this rare disease for better understanding of it and more qualified help for patients.

### Consent statement

Written informed consent was obtained from the patient’s legal guardians for publication of this case report. A copy of the written consent is available for review by the editor-in-chief of this journal.

## Abbreviations

MVK: mevalonate kinase
MKD: mevalonate kinase deficiency
MA: mevalonic aciduria
HIDS: hyperimmunoglobulinemia D syndrome
IgA: immunoglobulin A
IgD: immunoglobulin D
IL-1: interleukin 1
TNF-α: tumour necrosis factor
